# Clinically‐Relevant Static Magnetic Field Induces Release of Encapsulated Molecules from Magnetoliposomes

**DOI:** 10.1002/smll.202511451

**Published:** 2026-03-02

**Authors:** Jules Mistral, Nadège Milhau, Didier Pin, Olivier Chapet, Paula Nunes De Oliveira, Anatoli Serghei, Guillaume Sudre, Catherine Ladavière, Laurent David

**Affiliations:** ^1^ UMR 5223, Ingénierie des Matériaux Polymères (IMP) Université Claude Bernard Lyon 1, INSA Lyon Université Jean Monnet CNRS Villeurbanne France; ^2^ Veterinary School of Lyon (VetAgro Sup) UPSP 2016.A104 Interactions Cellules Environnement Marcy l'Etoile France; ^3^ Department of Radiation Oncology CH Lyon‐Sud Pierre‐Bénite France

**Keywords:** liposomes, magnetically‐controlled drug release, magnetoliposomes, SPION, superparamagnetic iron oxide nanoparticles

## Abstract

Magnetoliposomes (MLs) are known for their great potential in drug release under an alternating current (AC) magnetic field (MF). However, AC magnetic fields require specific setups that remain poorly implemented worldwide, contrary to direct currents (DC) that are present in widely used magnetic resonance imaging (MRI) devices. In this work, we evidence the feasibility of a drug release triggered by a 1.5 T constant MF, currently used in clinics. The structuration of magnetite nanoparticles (NPs) stabilized by surface adsorption of citric acid (Fe_3_O_4_:CA NPs), and an optional chitosan shell (Fe_3_O_4_:CA:CS NPs) to enhance cytocompatibility, was followed under a 1.5 T constant by small‐angle X‐ray scattering (SAXS), and dynamic light scattering (DLS). MLs encapsulating both Fe_3_O_4_:CA NPs and fluorescent carboxyfluorescein (CF), were also submitted to 1.5 T, and the CF fractional release was monitored. The effect of Fe_3_O_4_:CA:CS NPs addition externally to MLs in the suspension was investigated. Results evidenced that both Fe_3_O_4_:CA and Fe_3_O_4_:CA:CS NPs formed necklace‐like aggregates under DC MF. Fluorescence experiments demonstrated a significant CF release from MLs, which was enhanced by the addition of external NPs, while empty liposomes exhibited negligible releases. Finally, a biological evaluation of MLs and NPs revealed an excellent cytocompatibility.

## Graphical abstract

This work demonstrates the feasibility of a molecule release from magnetoliposomes triggered by a constant 1.5 T magnetic field currently used in clinical settings.

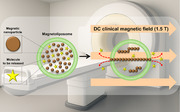

## 1 Introduction

Liposomes have numerous applications as drug carriers, with many assets such as biodegradability, biocompatibility, and low immunogenicity [1, 2, 3, 4]. Additionally, their unique structure, consisting in a spherical lipid bilayer with an internal aqueous cavity, enables the encapsulation of both hydrophilic and/or hydrophobic substances [5, 6, 7]. Also, the variety of lipid formulations allows tuning their physicochemical properties, such as surface charge, colloidal stability, or phase transition temperature (*T*_m_) [8, 9]. For all these reasons, liposomes have received major interest for decades, and numerous liposome‐based therapeutics are today approved by the EMA and FDA [10, 11].

Drug delivery from liposomes can be triggered through various stimuli, such as pH, enzymes, ultrasounds or light [12, 13, 14]. Another strategy is the encapsulation of superparamagnetic iron oxide (SPION) nanoparticles (NPs) within liposomes, enabling a drug release triggered by an external magnetic field (MF) [15, 16, 17]. Such systems with NPs located either in the lipid bilayer or in the aqueous cavity are called magnetoliposomes (MLs) [18]. Several works reported a drug release from MLs under an alternating current (AC) MF, using the magnetic hyperthermia effect [19, 20, 21, 22]. Indeed, NPs submitted to alternating MF generate heat through Néel (magnetic moment reversal) and Brownian (particle rotation) relaxations [23]. This increase of temperature above the phase transition temperature of lipids considerably enhances the permeability of the lipid bilayer and ultimately leads to the release of the encapsulated substance [24].

When they are not encapsulated within liposomes, magnetic NPs used for biomedical applications are often coated with a polymer shell to improve their biocompatibility. Among the variety of reported coatings, chitosan (CS) has been widely employed [25, 26]. Indeed, in addition to cytocompatibility enhancement, CS offers functional groups for further biological modifications or drug loading [27, 28]. However, the use CS coated NPs for therapeutic applications in an MF environment has been restricted to magnetic hyperthermia [29, 30], while the possibility of taking advantage of these systems to trigger and/or enhance a drug release under direct current (DC) MF has not been studied so far.

As mentioned previously, AC MF‐triggered drug release has been studied in several works. However, this strategy suffers from low implementation of clinical hyperthermia [31, 32]. Indeed, this technique still faces major economic and technical hardships, such as a high cost, poor focusing and non‐homogeneous heating, and a lack of protocol standardization [33, 34]. In contrast, direct current (DC, and not AC) based clinical devices such as MRI have been used worldwide for decades. Thus, beyond fundamental differences in triggering mechanisms, the main advantage of DC magnetic field–based strategies lies in their direct compatibility with existing clinical infrastructure, without the need for additional hardware or localized heating. Therefore, a strategy founded on the use of a static magnetic field as the stimulus for drug release appears much more suitable, while relying on a different triggering mechanism from MLs. In the DC (constant field) configuration, the drug release would occur through internal NP structuration, ultimately leading to bursting or perforation of ML lipid membranes. We identified several works reporting magnetic NPs forming small aggregates under DC MF [35, 36, 37, 38, 39]. However, MF strengths investigated were often rather low compared to clinical fields, and the scope was restrained to bare NPs instead of MLs. To the best of our knowledge, only one group studied the effect of DC MF on the morphology of giant polydisperse MLs with diameters of 30 µm [40]. However, such diameters are too high for in vivo applications, including drug delivery, for which optimal liposome sizes are reported to lie between 100 and 200 nm [41, 42, 43]. Finally, we could not find any previous study showing a drug release from MLs triggered by a DC MF.

Thus, this work aims to study the possibility and the efficiency of a drug release triggered by a DC MF. To this end, we firstly synthesized magnetite (Fe_3_O_4_) superparamagnetic nanoparticles (NPs) by the co‐precipitation method [44]. These NPs were subsequently stabilized in surface by the adsorption of citric acid molecules (CA), and they were encapsulated into the aqueous cavity of liposomes to form systems referred to as “magnetoliposomes” (MLs). To study and monitor the release from MLs, carboxyfluorescein (CF) was co‐encapsulated with magnetic NPs as a fluorescent probe. This fluorophore was used in several anterior studies as a model probe [20, 45, 46]. Indeed, it does not interact with lipid membranes, which makes it ideal to monitor precisely the release from MLs [47]. In addition, the effect on CF release of adding external Fe_3_O_4_:CA NPs with a CS shell was investigated.

This study is divided in two main parts. Firstly, we study the structuration of Fe_3_O_4_:CA and Fe_3_O_4_:CA:CS NPs submitted to a DC MF (*B*_0_ = 1.5 T, a value largely used in clinics in MRI devices). Secondly, we evaluate the CF release from various MLs suspensions submitted to *B*_0_, deciphering a possible release scenario.

## 2 Experimental Part

### 2.1 Materials

Iron (II) chloride tetrahydrate (FeCl_2_·4H_2_O, purity ≥ 99%), iron (III) chloride hexahydrate (FeCl_3_·6H_2_O, purity ≥ 99%), ammonium hydroxide (28%‐30%), citric acid (CA, 99%), sodium carbonate, sodium bicarbonate, acetic acid, ammonium acetate, propylene glycol, acetic anhydride, Dulbecco's Phosphate Buffered Saline (PBS), 5(6)‐carboxyfluorescein, and Triton X100 were purchased from Sigma–Aldrich (France). Hydrochloric acid (HCl, 37%), chloroform, and sodium chloride were obtained from Carlo Erba Reagents (France). Ultrapure water was obtained from Aguettant (France). Commercial chitosan (CS) with a low degree of acetylation (DA) from shrimp shell (batch type 244/020208; DA < 1%; $\bar{M_{w}}$ = 186 kg/mol; $\bar{M_{n}}$ = 97 kg/mol; *Đ* = 1.9) was bought from Mahtani chitosan Pvt. Ldt (Veraval, India). 1,2‐dipalmitoyl‐*sn*‐glycero‐3‐phosphocholine (DPPC, C_40_H_80_NO_8_P), 1,2‐dipalmitoyl‐3‐trimethylammonium propane (DPTAP, C_38_H_76_NO_4_Cl), and 1,2‐dipalmitoyl‐*sn*‐glycero‐3‐phosphoethanolamine‐*N*‐[methoxy(polyethyleneglycol)‐5000] (DPPE‐PEG5000, C_265_H_531_NO_123_P), extrusion kit, and polycarbonate membranes (with 400 nm pore size) for magnetoliposome elaboration were purchased from Avanti Polar Lipids Inc. (USA). The dialysis membrane with a molecular weight cut‐off (MWCO) of 3.5 kDa used for the purification of NPs was purchased from Spectrum Laboratories (USA). D‐Tube dialyzers Maxi (dialysis cassettes) with a MWCO of 12–14 kDa for the CF release studies were obtained from Millipore (France).

For cytotoxicity analysis, 25 cm^2^ and 75 cm^2^ Falcon flasks were obtained from VWR International (France). Dulbecco's Modified Eagle's Medium (DMEM) supplemented with 20% fetal calf serum, 2 mm l‐glutamine, and 2% penicillin/streptomycin/amphotericin B was purchased from Eurobio (France), as well as 0.25% trypsin/EDTA. CCK8 assay kits were supplied by Sigma–Aldrich (France).

### 2.2 Depolymerization and Reacetylation of Chitosan

The initial low DA CS was successively depolymerized and reacetylated following a procedure described elsewhere [48, 49], yielding $\bar{M_{w}}$ of 80 kg/mol, *Đ* = 1.6, and DA of 14%. 20 g of CS ($\bar{M_{w}}$ = 600 kg/mol, DA ∼ 1%) were dissolved, and stirred during 24 h in 4 L of a 0.2 m acetic acid/0.15 m ammonium acetate buffer (pH = 4.5). The depolymerization process was initiated by the addition of 77 mL of sodium nitrite solution at 1 mg/mL. According to existing depolymerization curves [48], the reaction was stopped after 55 min by the addition of ammonium hydroxide until reaching a basic pH (∼10–11) to precipitate CS. The obtained CS was washed and centrifuged several times (10 min, 4000 g, *T* = 10°C) until reaching neutral pH (∼6.3), and was subsequently freeze‐dried. For the reacetylation, 6 g of depolymerized low DA CS were dissolved in a propylene glycol/water mixture (50/50 v/v) with acetic acid at 3.3 g/L. A desired amount of acetic anhydride to get a DA of ∼ 15% was added and the solution was stirred overnight. The reaction was stopped in the same manner as depolymerization. CS was washed, centrifuged (10 min, 4000 g, *T* = 10°C), and freeze‐dried. CS $\bar{M_{w}}$ was evaluated by SEC [50], and its DA was measured by ^1^H NMR using the Hirai method [51].

### 2.3 Synthesis of Fe_3_O_4_:CA Nanoparticles

Bare magnetite Fe_3_O_4_ NPs were first synthesized by Fe(II)‐Fe(III) co‐precipitation. 0.905 g of FeCl_3_·6H_2_O and 0.333 g of FeCl_2_·4H_2_O (stoichiometric ratio 2:1) were dissolved in 100 mL of deionized water with 310 µL of HCl. The solution was placed under an argon atmosphere, heated to 60°C, and stirring was set at 80–90 rpm. After 1 h, stirring was increased to 170–180 rpm, and 15 mL of ammonium hydroxide were added dropwise. Stirring was maintained for 30 min, and the temperature was increased to 90°C. Then, 0.967 g of CA was added (post‐precipitation) to the solution corresponding to a Fe/CA molar ratio of 1 to provide a colloidal stabilization to NP suspensions by electrostatic repulsion between their surfaces. After 90 min, heating and stirring were stopped. Fe_3_O_4_:CA NPs were collected by magnetic decantation. Supernatant was discarded, and 5 mL of deionized water were added to re‐disperse the NPs. Particles were subsequently dialyzed 24 h against 6 different baths of deionized water (dialyzate/bath volume = 1/200) using a 3.5 kDa membrane to reach neutral pH and leak out the CA excess. The obtained Fe_3_O_4_:CA NPs were observed by TEM. Their diameter histograms, adjusted with a log‐normal law, indicated an average diameter of 10.0 ± 2.2 nm (Figure SI1).

### 2.4 Synthesis of Fe_3_O_4_:CA:CS Nanoparticles

26 mg of CS with a DA of 14% were dissolved in 13 mL of a HCl acid solution at pH = 2.2, in order to yield a 2% (w/w) CS solution. Stirring was kept at 400 rpm overnight. Then, 6 mL of a Fe_3_O_4_:CA NP suspension at a concentration of 6.5 mg/mL was introduced in a 60 mL glass vial. Mechanical stirring was set at 350 rpm, and the CS solution was added dropwise to the NP suspension. After 30 min, 19 mL of PBS 1X buffer were added dropwise (1/2 volume ratio to total suspension) to adjust the pH of the suspension to ∼ 6.8, thus allowing for partial deprotonation and adsorption of CS chains onto the Fe_3_O_4_:CA NP core. Stirring was kept for 15 min. Then, the resulting Fe_3_O_4_:CA:CS NP suspension was washed 4 times with deionized water and particles were collected with a permanent magnet. A 1 mL aliquot of the final NP suspension was collected, placed in an oven at 110°C, and weighed after water evaporation to evaluate the weight concentration of the suspension. Finally, Fe_3_O_4_:CA:CS NPs were sonicated with an ultrasonic probe (Sonics VC 505 Vibra Cell equipped with tapered microtip) for 20 min at an amplitude of 30% in order to reach an optimal dispersion prior to their use. Dynamic light scattering (DLS) measurements in water indicated an average hydrodynamic diameter (*D_Z_*) of 160.6 ± 2.6 nm, and a polydispersity index value (PDI) of 0.14 ± 0.02 for Fe_3_O_4_:CA:CS NPs. The inversion of the ζ‐potential of Fe_3_O_4_:CA:CS NPs (+ 38.1 ± 4.1 mV) compared to Fe_3_O_4_:CA NPs (−46.3 ± 14.3 mV) indicated the effective cationic CS coating, as shown in Figure SI2.

### 2.5 Elaboration and Purification of Magnetoliposomes Loaded with Fe_3_O_4_:CA Nanoparticles

MLs only loaded with Fe_3_O_4_:CA NPs were elaborated for characterization purposes (cryo‐TEM observations), and MLs loaded with both Fe_3_O_4_:CA NPs and CF were designed for CF release investigations under a DC MF. In both cases, ML suspensions with a lipid concentration of 10 mm were obtained by the film hydration method [52]. The desired amounts of DPPC, DPPE‐PEG 5000, and DPTAP lipids were dissolved in chloroform (CHCl_3_) at molar fractions of DPPC/DPPE‐PEG 5000/DPTAP 90/5/5, and CHCl_3_ was evaporated at 60°C. The resulting lipid film was hydrated for 20 min at 60°C, and the ML suspensions were extruded 11 times at 70°C through a polycarbonate membrane with pore diameters of 400 nm. Unencapsulated Fe_3_O_4_:CA NPs and empty liposomes were respectively removed by salt‐induced aggregation (SIA) and magnetic chromatography (MC), following a procedure described and validated elsewhere [53]. Typical cryo‐TEM images are shown in Figure SI3. It was not possible to prepare such MLs loaded with bare Fe_3_O_4_ NPs, *i.e*., without the CA coating, due to the formation of NP aggregates.

For MLs loaded with Fe_3_O_4_:CA NPs, the lipid film hydration was performed with 800 µL of a Fe_3_O_4_:CA NP suspension at an initial concentration of 31.3 mg/mL, and 200 µL of deionized water. For MLs encapsulating Fe_3_O_4_:CA NPs and CF, the lipid films (9.8 mg of lipids for each film) were hydrated with 800 µL of Fe_3_O_4_:CA NPs at an initial NP concentration of 31.3 mg/mL, and 200 µL of 0.1 m CF solution, both diluted in a carbonate buffer (0.05 m, pH 9.2, ionic strength 0.2 m). After the extrusion step, the excess CF was removed by successive dialysis (MWCO 12–14 kDa, dialysate/bath volume ratio = 200) in carbonate buffer (0.05 m, pH 9.2) prior proceeding to purification by SIA and MC. In both cases, the final concentration of the NP suspension was 25 mg/mL after water or CF addition.

Finally, liposomes loaded only with CF were also prepared. The elaboration was carried out following the same protocol as the one described above for MLs loaded with NPs and CF, except that the hydration of the lipid film was performed with 800 µL of deionized water instead of a NP suspension, and 200 µL of 0.1 m solution of CF in carbonate buffer (0.05 m, pH 9.2).

### 2.6 Evaluation of Size, Size Distribution, Surface Charge of Nanoparticle and Magnetoliposome Suspensions by Dynamic Light Scattering (DLS), and Zetametry

DLS and zeta potential measurements were conducted to determine the average hydrodynamic diameter (*D_Z_*), polydispersity index (PDI), and zeta potential (ζ) of NPs and MLs using a Zetasizer Nano ZS (Malvern Instruments, U.K.). Measurements were performed at 25°C, either in deionized water or in carbonate buffer (0.05 m, pH 9.2), depending on the type of sample. The refractive index and viscosity of both aqueous media were set to 1.33 and 0.8904 cP, respectively. NP and ML suspensions were diluted before being analysed to reach concentrations of 0.3 g/L and 0.3 mm, respectively. Three measurements were performed and averaged for each sample. For DLS measurements, the laser operated at a wavelength of 633 nm, with a measurement angle of 173°C. Zeta potential (ζ) value was obtained from the measurement of the electrophoretic mobility (µ_*e*_), according to Smoluchowski's Equation (1):

(1) $$\mu_{e}=\frac{\varepsilon_{0}\varepsilon_{r}}{\eta }\;\zeta$$

where *η*, ε_0_ and ε_*r*_ are the medium viscosity, permittivity of vacuum, and relative permittivity of the measurement medium, respectively.

### 2.7 Evaluation of Nanoparticle Phase Composition by Wide Angle X‐Ray Scattering (WAXS)

To evaluate the phase composition of synthesized NPs, WAXS measurements were carried out at the European Synchrotron Radiation Facility (ESRF, Grenoble, France) on the D2AM beamline. For these experiments, NPs, synthesized following the protocols described above, were freeze‐dried to obtain powders that were sandwiched between two Kapton films and mounted on a custom‐designed holder. The incident photon energy was 16.000 keV. Scattered intensities were collected using a 2D WAXS Open for SAXS (WOS) detector (IMXPAD, France). The sample‐to‐detector distance was set to ∼ 10 cm. Calibration of the scattering vector *q* was performed using a chromium oxide (Cr_2_O_3_) powder standard. All scattering patterns were normalized to the transmitted beam intensity, and azimuthal averaging was performed with respect to the beam center. Background contributions from an empty holder containing only the Kapton films were recorded separately and subtracted from each dataset to obtain the final scattering profiles, which are available in Figure SI4.

### 2.8 Evaluation of Nanoparticle Structuration Under External DC Magnetic Fields of Various Strengths by Small Angle X‐Ray Scattering (SAXS)

**FIGURE 1:**
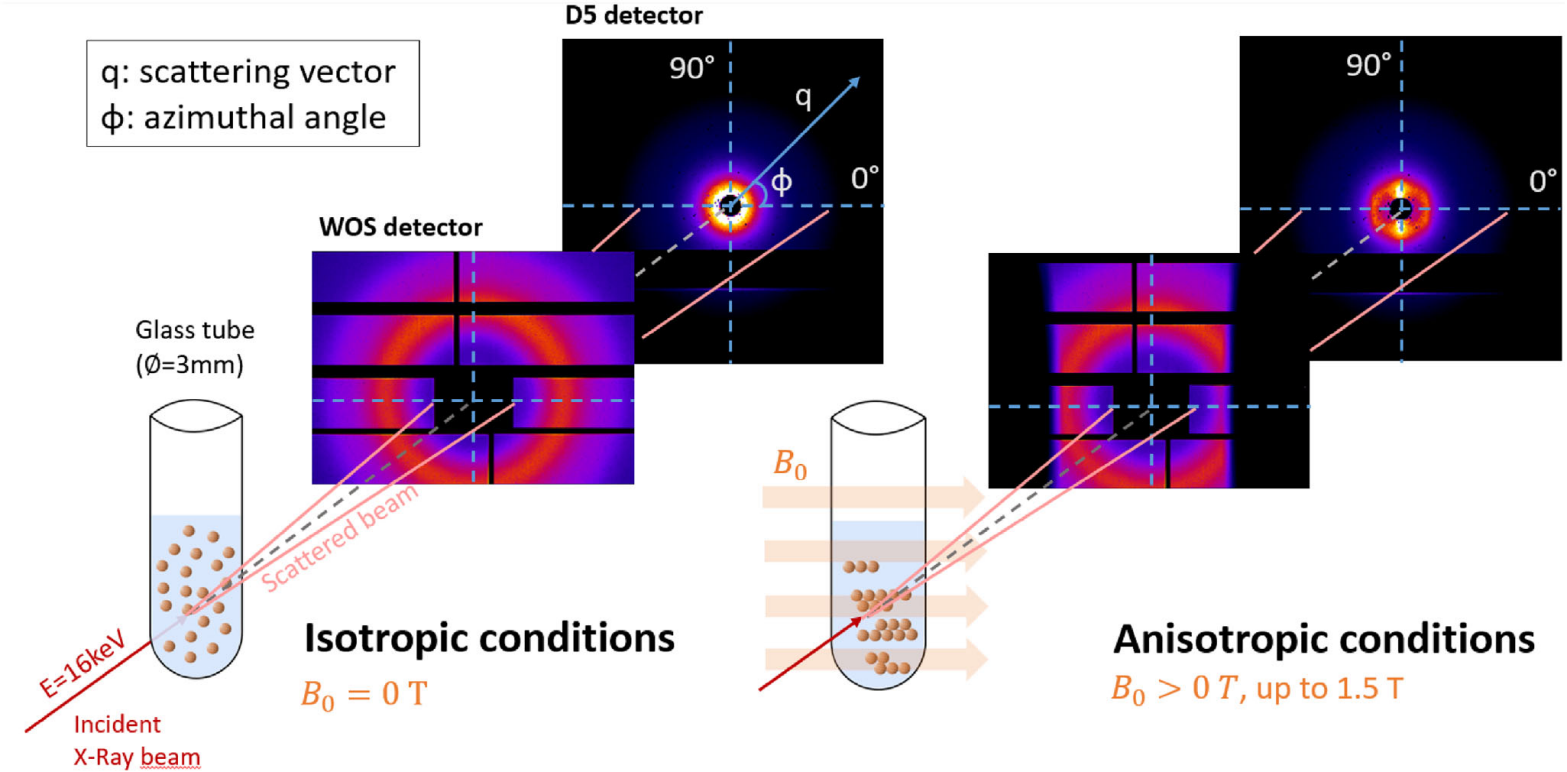
Schematic illustration of the SAXS/WAXS setup used to investigate NP structuration under a DC MF. NPs contained in a glass capillary (Ø = 3 mm) exhibit isotropic scattering at B_0_ = 0 T and anisotropic scattering upon application of a DC MF (B_0_ up to 1.5 T). SAXS patterns were collected with the D5 detector, while a WOS detector placed upstream enabled simultaneous WAXS acquisition. The scattering vector q and azimuthal angle φ are indicated.

SAXS experiments were also performed at ESRF on D2AM beamline, using the same beam in the conditions of simultaneous SAXS/WAXS experiments, in order to characterize the microstructure of NP suspensions in the presence of an external DC MF (*B*_0_) of variable amplitude. The suspensions were prepared in ultrapure water at concentrations of 1 mg/mL, and were sonicated during 20 min before being transferred in glass tubes (Deutero GmbH, ref. 600020–200, external diameter: 3 mm, length: 60 mm, width: 0.3 mm). The external DC MF was generated using an electromagnet TE2M 50 (TE2M, France). A home‐made tube holder was designed and inserted in the electromagnet device so that the incident beam could cross the NP suspension, as shown in Figure SI5. The sample to detector (D5 solid state detector, IMXPAD, France) distance was set to 3.06 m. Again, the *q*‐range calibration was performed using a silver behenate (AgBe) standard. For each sample, measurements were performed at 15 different *B*_0_ amplitudes in the range [0 – 1.5 T], increasing and then decreasing *B*_0_ to evaluate the reversibility of the nanostructuration. Thus, an analysis cycle for a given sample consisted in 29 different measurements. For each *B*_0_, the scattering images were acquired during 50 s. After radial averaging in 48 sectors, the obtained scattering diagrams were normalized by transmission, and the signal of the empty cell (solvent + tube) was subtracted in order to obtain the net NP signal. The treatment of 2D reconstructed images from the SAXS D5 detector was also performed, using ImageJ software. Brightness and contrast were adjusted at identical values for all the samples so that the images could be qualitatively compared. The principle of our in situ SAXS experiments is illustrated in Figure 1.

### 2.9 Exposure of Liposome and Magnetoliposome Suspensions to DC Magnetic Fields for Release Studies

500 µL of a liposomal or magnetoliposome suspension encapsulating CF were introduced in a 2 mL glass vial. The home‐made tube holder (empty) was placed on the electromagnet (Figure SI3). The distance between the 2 polar nuclei was adjusted using a Gaussmeter (Hirst Magnetics, U.K.) to reach *B*_0_ = 1.5 T with a current of 6 A applied by a current generator. Then, 100 µL of the suspension were introduced in the glass tube (Deutero GmbH), and the current was turned on for 25 min. The liposome or magnetoliposome suspension was then withdrawn from the glass tube and transferred in a second 2 mL glass vial. This operation was repeated 5 times, generating 5 different samples, until the 500 µL of initial suspension contained in the first vial were exposed to MF, and transferred to the second vial after pooling the five tube samples. Indeed, it was not easy to perform the experiment on the volume of 500 µL in one go, because of (*i*) the small volume of the glass tubes, (*ii*) the small area of the polar nuclei which implied a reduced zone of MF application, (*iii*) the reduced space (5 mm) needed between the 2 polar nuclei to reach *B*_0_ = 1.5T, so that only tubes with external diameters of ∼ 3 mm could be used.

### 2.10 Evaluation of CF Release from Liposome and Magnetoliposome Suspensions Using Fluorescence Spectroscopy

50 mL of carbonate buffer (0.05 M, pH 9.2), and a magnetic stirrer were introduced in 2 different burettes of 100 mL. Then, 450 µL of the MF‐exposed liposome or magnetoliposome suspension, referred here as DC sample, were added in a 12–14 kDa MWCO dialysis cassette. Another 450 µL of the initial suspension, which had not been submitted to MF, were introduced into a second cassette as a control sample. Both cassettes were simultaneously introduced in each burette. Magnetic stirring was turned on and set to 450 rpm at room temperature. At given time points, the dialysate was collected, and fluorescence intensity was measured using a SynergyMX fluorimeter (BioTek, USA). The excitation and emission wavelengths were set at 493 and 517 nm, respectively. 5 different measurements were performed and averaged for each point. After 24 h, 1.5 mL of Triton X100 (3% V/V) were added to the dialysis bathes in order to leak out all CF after diffusion of Triton X100 in the cassette, and by disrupting the lipid membranes of liposomes or MLs. Finally, fluorescence intensity was measured again 24 h after the addition of Triton X100. Assuming all CF was released after disruption of MLs, the fractional release (FR) was calculated with the following Equation (2):

(2) $$R=\frac{I_{\mathrm{MLs}}\left(t\right)-I_{\mathrm{buffer}}}{I_{\max}-I_{\mathrm{buffer}}}\;$$

Where *I*_MLs_, *I*_buffer_, and *I*_max_ are the fluorescence intensities of the dialysis bath, the buffer (carbonate 0.05 M, pH 9.2), and the dialysis bath 24 h after Triton X100 addition, respectively.

### 2.11 Cytotoxicity Experiments of Fe_3_O_4_:CA:CS Nanoparticles

The cytotoxicity of Fe_3_O_4_:CA:CS NPs synthesized in this work was evaluated following a procedure described in previous works [53, 54]. Briefly, canine dermal fibroblasts were isolated and cultured from the abdominal surface of a dead neonatal dog obtained in VetAgro Sup (Marcy l'Etoile, France), in accordance with its ethical committee (agreement number 1455). Cell viability was investigated following the international standard operation (ISO) 10993–5, “Tests for in vitro cytotoxicity”, using the CCK‐8 assay according to the manufacturer's instructions. For this, fibroblasts were seeded at a density of 1 × 10^4^ cells/ well in a 96‐well plate. After 2 days of culture, they were exposed to NPs for durations up to 72 h with concentrations in the range [0‐1] mg/mL for NPs. After incubation, 10 µL of CCK8 were added into each well and kept at 37°C for 2 h. The absorbance at 450 nm was determined using a Multiskan microplate reader (Thermofisher, USA). Cell viability was determined as the ratio of the optical density (OD) of exposed cells to the OD of the untreated cells x100.

## 3 Results and Discussion

### 3.1 Evidence of Superparamagnetic Nanoparticle Structuration Under an External DC MF

#### 3.1.1 SAXS Study of the Superparamagnetic Nanoparticle Structuration and Its Reversibility

**FIGURE 2:**
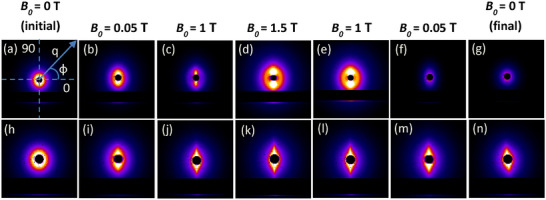
SAXS experimental scattering patterns at different B_0_ values for (a–g) Fe_3_O_4_:CA NPs and (h–n) Fe_3_O_4_:CA:CS NPs. The direction of the applied field B_0_ is horizontal.

As described above, two types of magnetite superparamagnetic NPs were synthesized in this work: *i*) Fe_3_O_4_ NPs with citric acid molecules chemisorbed on their surface (Fe_3_O_4_:CA NPs) and *ii*) the same coated with CS chains (Fe_3_O_4_:CA:CS NPs). Figure 2 displays 2D‐SAXS scattering patterns of Fe_3_O_4_:CA NPs (a‐c) and Fe_3_O_4_:CA:CS NPs (d‐f) at different stages of the measurement cycle (ascending and descending from *B_0_* = 0 to 1.5 T, where the magnetic field is oriented horizontally). The complete sets of 25 scattering patterns for each of these samples are given in supplemental Video (SV1) under the form of animated movies. As expected, both initial patterns were isotropic, reflecting the random dispersion of NPs in suspension in the glass tubes. At the highest *B_0_* (1.5 T), patterns were on the contrary, strongly anisotropic, with high scattering intensities spreading in the vertical direction. This was indicative of the formation of aggregates of NPs extending in the horizontal field axis direction in the glass tube in the form of extended necklace‐like aggregates of particles, and, at a larger length scale, to microfibrils oriented in the horizontal direction, as reported in previous studies [35, 36]. Interestingly, this NP structuration occurred progressively and started at low *B*_0_ values (0.05 T) for the most elementary objects. Also, the final scattering patterns at *B*_0_ = 0 T were very different from the initial ones, and the presence of a chitosan coating largely impacted the final morphology at zero field. Indeed, Fe_3_O_4_:CA NP suspension presented a weak but isotropic pattern, showing partial reversibility of the magnetic structuration. This was coherent with their intrinsic superparamagnetic behavior [54], as Fe_3_O_4_ NPs of 20 nm and below can be seen as single magnetic dipoles being either aligned with *B*_0_ or randomly oriented, depending on whether the field is turned on or off [55]. However, the signal intensity in the final isotropic configuration was quite low compared to the initial one, due to progressive sedimentation of aggregates of elementary particles, as shown by the evolution of the scattering patterns (see Figure 2; Movie SV1, and Figure SI6 showing aggregates in glass tubes at macroscopic scale). A very different behavior was observed for Fe_3_O_4_:CA:CS NPs, for which structuration was almost immediate and not reversible, probably because of CS coatings acted as “sticking layers”, and preserved the structure of micron‐range fibrils, aligned with *B_0_* axis (Figure SI6). Contrary to Fe_3_O_4_:CA NPs, the fibrillar structure prevented strong sedimentation after a decrease of *B*_0_. The slight rotation of the final scattering pattern may be due to the fall and tilt of the fibrillar structure when *B*_0_ was turned off, as shown in Figure SI6d. Therefore, an (unknown) part of the vertical scattering intensity may be due to surface reflection and diffraction from the fibrils formed under the MF.

**FIGURE 3:**
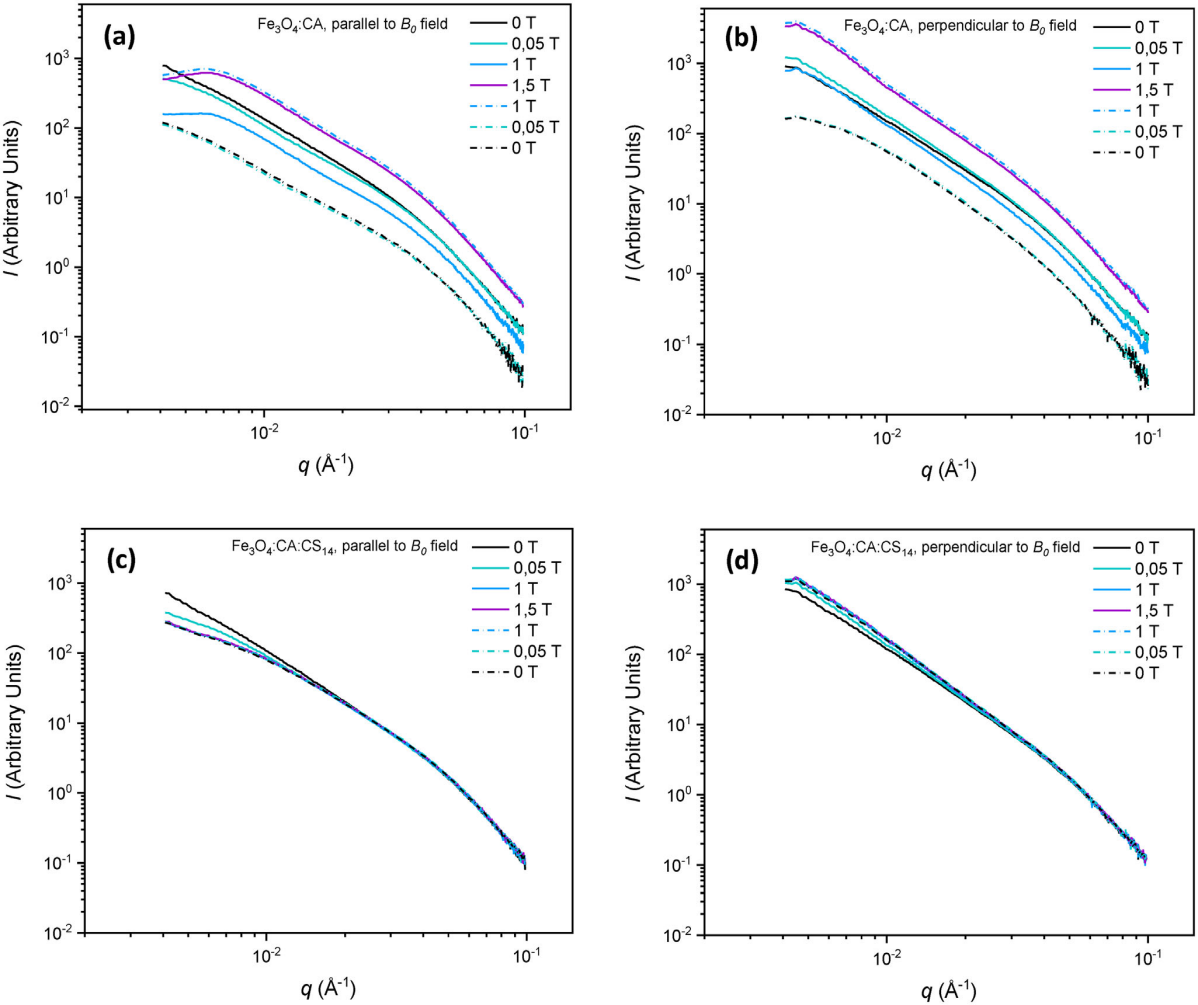
SAXS diagrams showing experimental data for different values of B_0_, for (a) Fe_3_O_4_:CA NPs (horizontal sectors, magnetic field direction), (b) Fe_3_O_4_:CA NPs (vertical sectors, perpendicular to field direction), (c) Fe_3_O_4_:CA:CS NPs (horizontal sectors), (d) Fe_3_O_4_:CA:CS NPs (vertical sectors). In a measurement cycle, B_0_ increases from 0 T to 1.5 T, and then decreases from 1.5 T to 0 T. Full lines and dashed lines on the graphs indicate data acquired during the ascending and descending phases of the cycle, respectively.

In addition, the scattered intensities in both horizontal and vertical directions, resulting from radial averages in angular sectors of 7.5° wide, were plotted as a function of the scattering vector *q*, for the same *B*_0_ than for the 2D patterns shown in Figure 2. Figure 3a,b displays the results for Fe_3_O_4_:CA NPs in the field direction and perpendicular to the applied field, respectively. The increase of *B*_0_ led to the appearance of a correlation peak particularly visible in the horizontal field direction at q* ∼7.10^−3^ Å^−1^, which was indicative of an inter‐distance of 2π/q*∼100 nm. However, this peak did not appear in the vertical direction, possibly due to reflection on the surface of fibrils. Since the elementary magnetite monocrystalline NPs exhibited a mean diameter close to 10 nm, we assume that the formation of necklaces/aggregates of less than 10 NPs could be an intermediate nano‐structuration level, the final auto‐association of such intermediate objects resulting in the formation of much larger fibrils (∼2 mm long, ∼300–400 µm in diameter, as shown in Figure SI6d). Indeed, this correlation peak started to appear at *B*_0_∼1 T and remained at the same *q* value until it disappeared below 1T. This revealed a nanostructure of micro‐fibrils resulting from the disordered arrangement of compact Fe_3_O_4_:CA NPs necklaces/aggregates with a size lower, but of the order of, 100 nm. Interestingly, Fe_3_O_4_:CA:CS NPs did not show exactly the same behavior. The scattered intensity at large *q* values was rather stable with variations of *B*_0_, showing a lesser impact of sedimentation. Differences in the scattered intensity could only be observed in the low *q*‐range. A scattered intensity decrease was observed in the horizontal direction upon application of the magnetic field, concomitant with the formation of the micro‐fibrils. The absence of a clear structure factor peak may be due to a coarser intermediate aggregate morphology in the presence of CS coating, promoting inter‐particular interactions, or a different structure of aggregates forming the fibrils (less compact, possibly with fractal morphology). Coherently with the 2D scattering patterns, the scattering intensity remained constant even during the decreasing phase of the *B*_0_ cycle, which confirmed that the structuration of Fe_3_O_4_:CA:CS NPs into microfibrils was not reversible, as a result of strung inter‐particle and aggregate interactions, possibly due to the CS coating inter‐diffusion.

#### 3.1.2 Influence of NP Concentration and Aqueous Suspension Medium on NP Magnetic Nanostructuration

**FIGURE 4:**
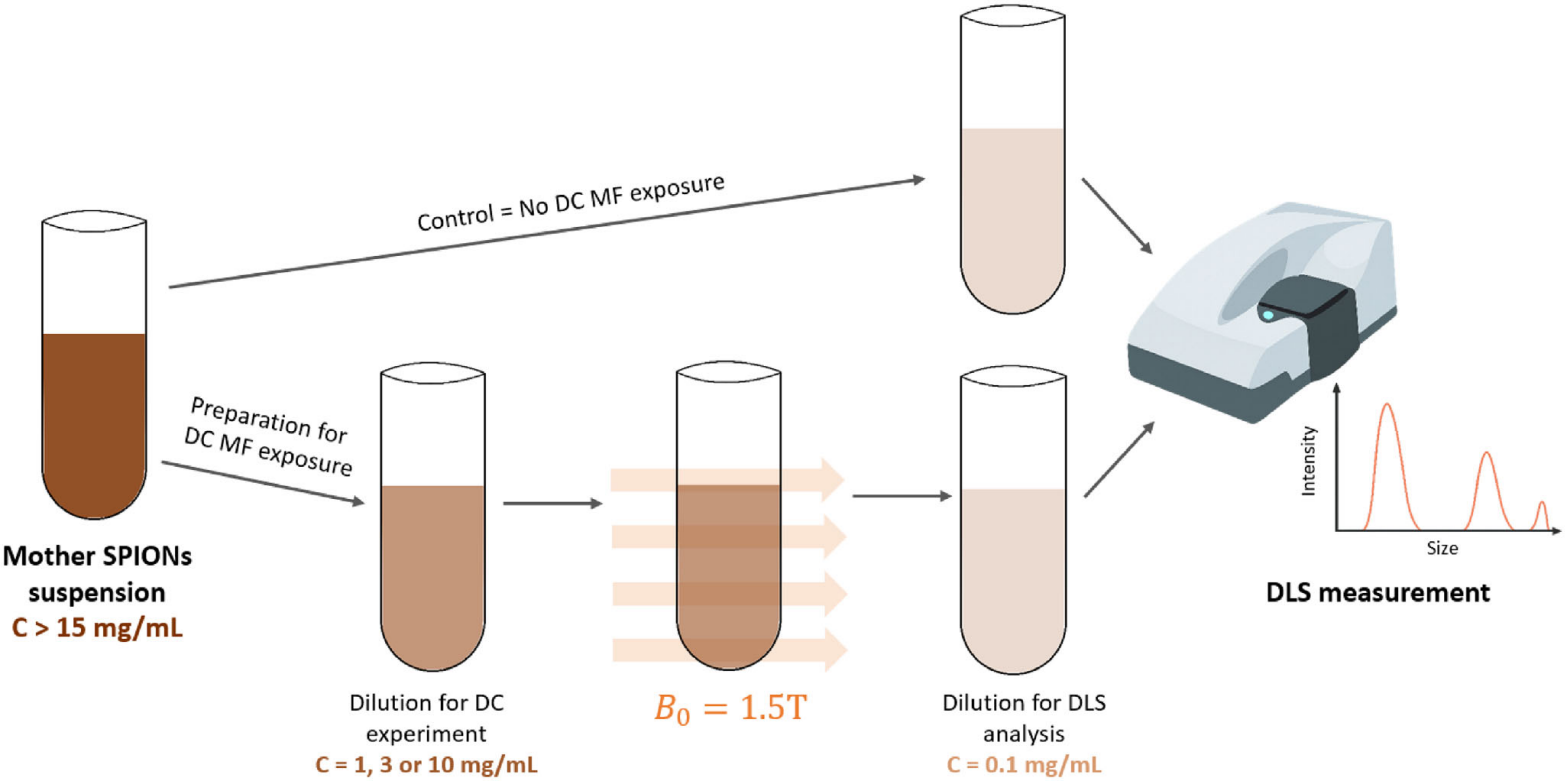
Illustration of the experimental principle used to assess by DLS the effects of DC magnetic field (DC MF) on NP structuration. Starting from a concentrated mother NP suspension, control samples are obtained by a single direct dilution to the DLS measurement concentration without magnetic field exposure. In contrast, field‐exposed samples undergo a first dilution to the target concentration for DC MF exposure (B_0_ = 1.5 T), followed by a second dilution to reach the DLS analysis concentration.

**FIGURE 5:**
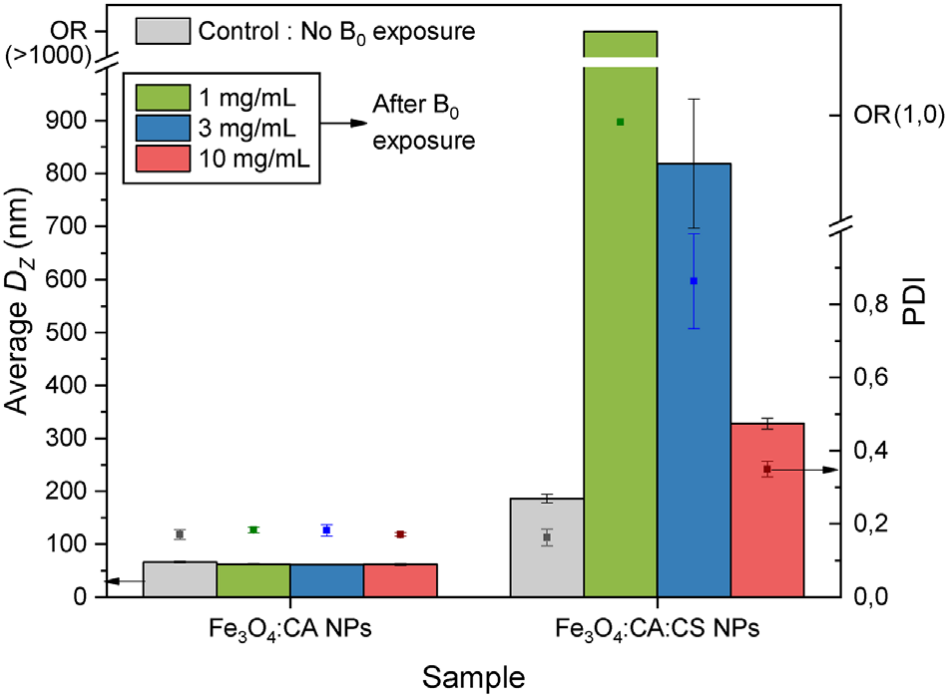
Influence of concentrations of Fe_3_O_4_:CA NPs and Fe_3_O_4_:CA:CS NPs suspended in water on average D_Z_ and PDI values, before (“control”) and after exposure to B_0_ = 1.5 T. Samples were diluted from a mother solution (C = 31.3 mg/mL for Fe_3_O_4_:CA NPs, and C = 15.2 mg/mL for Fe_3_O_4_:CA:CS NPs) to the desired concentration (1 mg/mL, 3 mg/mL or 10 mg/mL) for exposure to B_0_. They were subsequently diluted again to 0.1 mg/mL for DLS analysis. Samples referred as “Control” were not exposed to B_0_ and were directly diluted to 0.1 mg/mL. OR = D_z_ and PDI values out of the relevancy range, revealing a highly aggregated structure. Standard deviations were calculated on 3 different measurements.

In addition to SAXS experiments, DLS measurements were performed to study the evolution of average *D_Z_* and PDI values after an exposure to *B*_0_ = 1.5 T according to NP concentration, and aqueous suspension medium. The experiment principle is illustrated in Figure 4, and Figure 5 presents the results obtained at various NP concentrations in water. Coherently with SAXS experiments, the average *D_Z_* and PDI values of Fe_3_O_4_:CA NPs remained unchanged after exposure to DC MF, regardless of their concentration, evidencing partial reversible behavior in the aggregation/disaggregation process, although large aggregates may sediment and disappear from the studied volume. On the contrary, Fe_3_O_4_:CA:CS NPs were strongly affected by the magnetic field, with a permanent increase in *D_Z_* and PDI values, as a result of their “sticky coatings”. These changes were directly linked to their concentration in suspension. Unexpectedly, the largest structures were obtained with the lowest concentrations (1 mg/mL) with *D_Z_* > 1 µm, and PDI equal to 1.00. Such values lye out of the relevance range (OR) for DLS measurements, but they indicate a massive aggregation. Surprisingly, the size of aggregates decreased with the increase in NP concentration while remaining significant, even at the highest concentrations (10 mg/mL). Apparently, the highest NP concentrations are not optimal for aggregation: the magnetic field, produced by the denser superparamagnetic NP collections, could result in a lower effective magnetic field gradient, and thus less dragging force to associate NPs in pairs, multiple associations up to aggregates.

**FIGURE 6:**
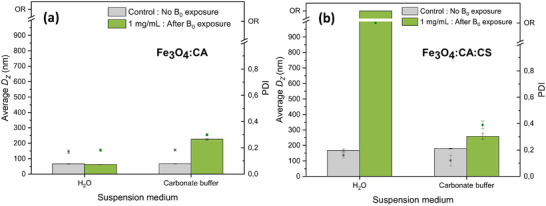
Influence of suspension medium (water or carbonate buffer 0.05 m, pH 9.2) on average D_Z_ and PDI values of (a) Fe_3_O_4_:CA NPs and (b) Fe_3_O_4_:CA:CS NPs, before (“control”) and after exposure to B_0_ = 1.5 T. Samples were diluted from a mother solution (C = 31.3 mg/mL for Fe_3_O_4_:CA NPs and C = 15.2 mg/mL for Fe_3_O_4_:CA:CS NPs) to the desired concentration (1, 3 or 10 mg/mL) for exposure to B_0_. They were subsequently diluted again to 0.1 mg/mL for DLS analysis. Samples referred as “Control” were not exposed to B_0_ and were directly diluted to 0.1 mg/mL. OR = D_z_ and PDI values out of the relevancy range, revealing a highly aggregated structure. Standard deviations were calculated on 3 different measurements.

Moreover, the suspension medium (water or carbonate buffer 0.05 m, pH 9.2) of NPs had a major influence on their structuration abilities. Figure 6a shows the results for Fe_3_O_4_:CA NPs, and Figure SI7 displays images of these NPs in glass tubes during *B_0_* exposure, both in water and carbonate buffer media. Fe_3_O_4_:CA NPs suspended in carbonate medium appeared to form aggregates visible to the naked eye, and de‐aggregation was not fully reversible. Indeed, their average *D_Z_* and PDI values were found higher after *B_0_* exposure. On the contrary, their structuration in water at the same concentration (1 mg/mL) was not directly visible but reversible, as average *D_Z_* and PDI values remained unchanged. This difference in aggregation behavior may be attributed to the ionic strength of the carbonate buffer (0.05 m, ionic strength 0.2 m, Debye length 7.2 nm), which prevents repulsive interactions between Fe_3_O_4_:CA NPs by screening citrate ions. This may ultimately favor NP aggregate formation under the external MF, and prevent de‐aggregation after removal the external magnetic field.

Regarding Fe_3_O_4_:CA:CS NPs, changing from an aqueous to the carbonate buffer (0.05 m, pH 9.2) interestingly led to an opposite effect (Figure 6b). While large irreversible structures were formed in water (average *D_Z_* and PDI values found to be OR), the carbonate buffer drastically disturbed the structuration, with moderate *D_Z_* and PDI increases after field exposure. This could be attributed to a neutralization and hydrophobization effect of CS chains at pH = 9, whereas a positive charge was carried by CS‐coated nanoparticles in water. Thus, in water, CS chains may keep a partial polycationic feature and an extended chain conformation, favoring inter‐chain interactions. Another possibility is to account for strong interactions between the amine groups of CS and CO_3_^2−^ carbonate ions possibly leading to CS carbamoyl polyanionic derivative at alkaline pH [56, 57, 58]. Again, this would lead to an additional electrostatic stabilization effect in the presence of CS and carbonate ions. Interestingly, *D_Z_* and PDI values of Fe_3_O_4_:CA NPs and Fe_3_O_4_:CA:CS NPs after *B_0_* exposure in carbonate buffer were found very close to each other, showing that the CS shell reorganization of NPs logically led to similar structural properties than for non‐coated Fe_3_O_4_:CA NPs.

Thus, the influence of the suspension medium on NP structuration highlights the importance of interparticle interactions in magnetically driven aggregation. While water and carbonate buffer were chosen here as model media, biologically relevant fluids such as serum could introduce additional effects, including the protein corona formation. These phenomena may alter NP structuration or modulate its kinetics under DC MF. Therefore, evaluating these systems in complex biological media represents a logical next step toward in vivo translation.

### 3.2 Evaluation of CF Release from MLs Under a 1.5T DC MF

**FIGURE 7:**
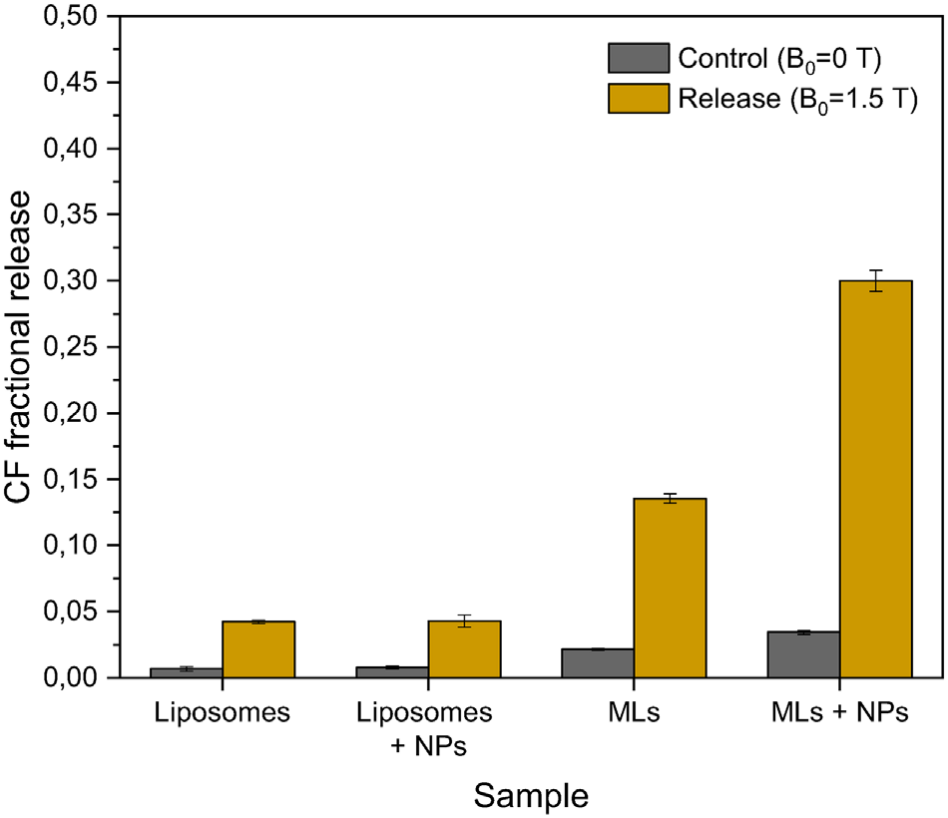
Comparison of CF fractional release (FR) for different samples after exposure to B_0_ = 1.5 T during 25 min. Experiments were performed in carbonate buffer (0.05 m, pH 9.2), and at room temperature. All samples had a lipid concentration of 10 mm. MLs had an internal Fe_3_O_4_:CA NPs concentration of 60 µg/mL. For samples “Liposomes + NPs” and “MLs + NPs”, external Fe_3_O_4_:CA:CS NPs were added at a concentration of 1 mg/mL.

Four different ML formulations with identical lipid composition (DPPC/DPPE‐PEG5000/DPTAP, 90:5:5 mol/mol/mol) were evaluated for their ability to release CF under DC external MF: (*i*) bare liposomes, (*ii*) liposomes with external Fe_3_O_4_:CA:CS NPs at a concentration of 1 mg/mL (“Liposomes + NPs”, Figure 7), (*iii*) purified MLs with internal Fe_3_O_4_:CA, (*iv*) purified MLs with internal Fe_3_O_4_:CA NPs and external Fe_3_O_4_:CA:CS NPs added at a concentration of 1 mg/mL (“MLs + NPs”, Figure 7). Figure 7 shows the CF fractional releases (FR) measured in the release plateau for each of them, compared to their corresponding controls which were not exposed to the external magnetic field *B*_0_. First, significant FR were observed for MLs and MLs + NPs, evidencing a major role of *B*_0_ exposure in CF release. On the basis of previous SAXS and DLS analyses, we suggest that the CF release is allowed by NP aggregation across the MLs membranes, leading to bursting or at least perforation of the lipid membranes. Also, CF FR value was significantly higher with the addition of external Fe_3_O_4_:CA:CS NPs to the ML suspension. Consequently, the presence of external NPs contributes to the ML breakage or perforation from external particles, and thus, facilitates the release. However, the addition of external NPs to “simple” liposome suspensions (with no encapsulated NPs) was absolutely inefficient and resulted in a very low FR. This result clearly underlines the crucial role of MLs in CF release and demonstrates that external NPs are efficient only when associated with internal NPs, the rupture of lipid membranes resulting from superparamagnetic NP interactions in separated compartments. Also, given that the FR of MLs is about half of MLs + NPs fractional release, MLs formulated with a too low internal NPs density may remain intact. Therefore, the NP loading capacity must be high enough to destroy/perforate the lipid membrane. Finally, it was observed that the CF FR value from “simple” liposomes was apparently higher than its corresponding control with no applied field, even though it remained rather low compared to MLs. This result may arise from the experimental procedure during which the suspensions are transferred through needles into glass tubes, a process during which some liposomes may be damaged.

**FIGURE 8:**
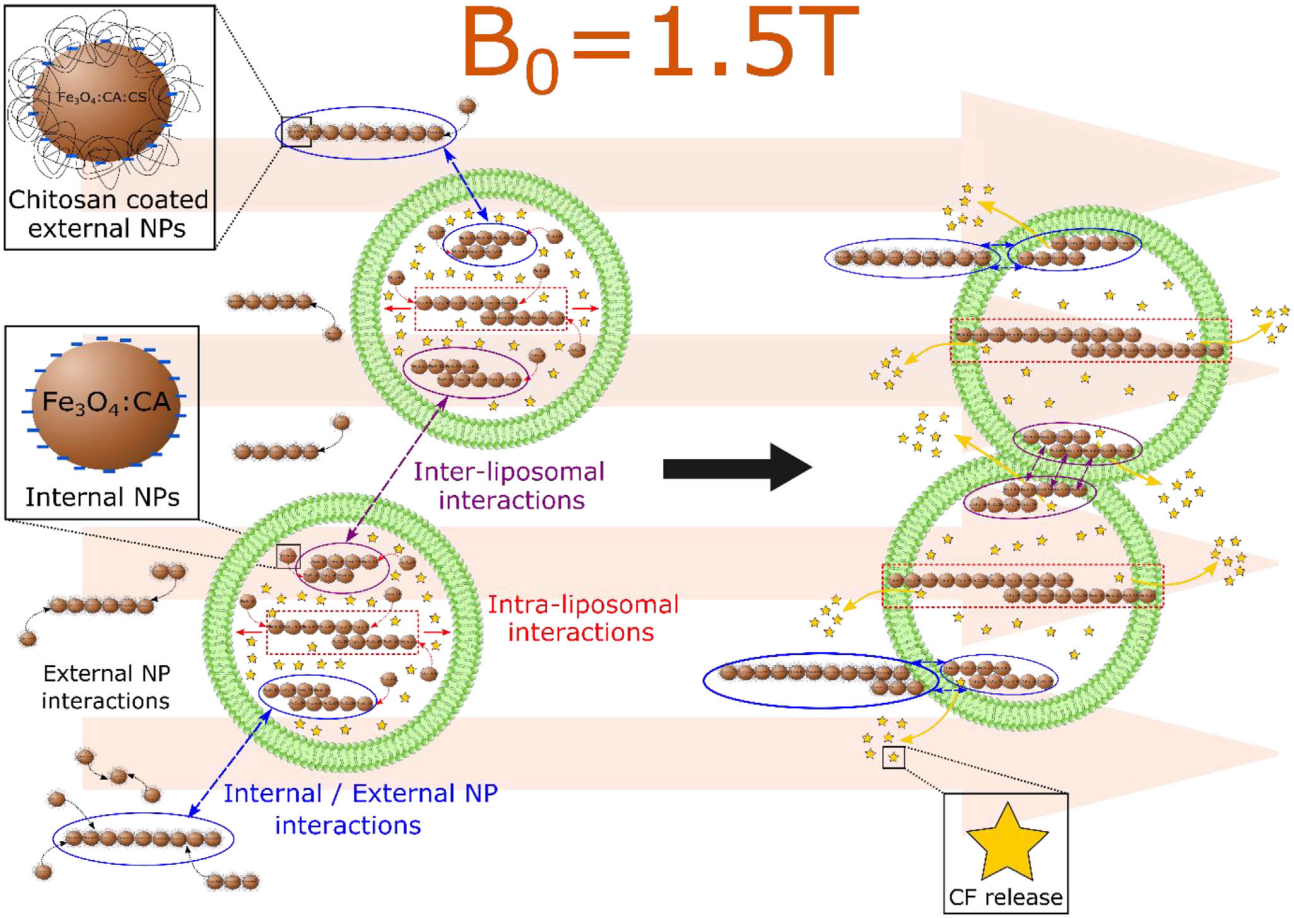
Illustration of the mechanisms involved in carboxyfluorescein (CF) release from magnetoliposomes (MLs) under a DC magnetic field (DC MF) (B_0_ = 1.5 T). Left panel: upon DC MF application, NPs organize into linear aggregates both inside the liposomes (red, “intra‐liposomal interactions”) and in the surrounding medium (black, external NP interactions). Interactions occur between aggregates confined within different liposomes (purple, “inter‐liposomal interactions”), as well as between internal and external NP aggregates (blue, “internal/external NP interactions”). Right panel: CF release from MLs, resulting from the combined actions of the mechanisms illustrated in the left panel.

As a conclusion, achieving a high DC MF‐induced release fraction requires several key design considerations, including (*i*) the use of small and CA–stabilized superparamagnetic iron oxide NPs, (*ii*) a high NP loading within MLs, and (*iii*) a carefully engineered formulation in terms of NP type, surface coating, concentration, and spatial localization, involving both internal and external NPs. In contrast to AC MF‐driven release involving thermal heating, we attribute CF release under DC MF to a combination of mechanically driven processes, illustrated on Figure 8. They include (*i*) intra‐liposomal Fe_3_O_4_:CA NP structuration, (*ii*) internal Fe_3_O_4_:CA / external Fe_3_O_4_:CA:CS NP interactions, and (*iii*) inter‐liposomal interactions between different chain‐like aggregates of Fe_3_O_4_:CA encapsulated NPs. Finally, the protocol presented in this work enables CF release by DC MF within 25 min, which corresponds to typical MRI examination durations, thereby enabling its application at a clinical stage and its combination with diagnostic imaging.

### 3.3 Cytocompatibility Evaluation

**FIGURE 9:**
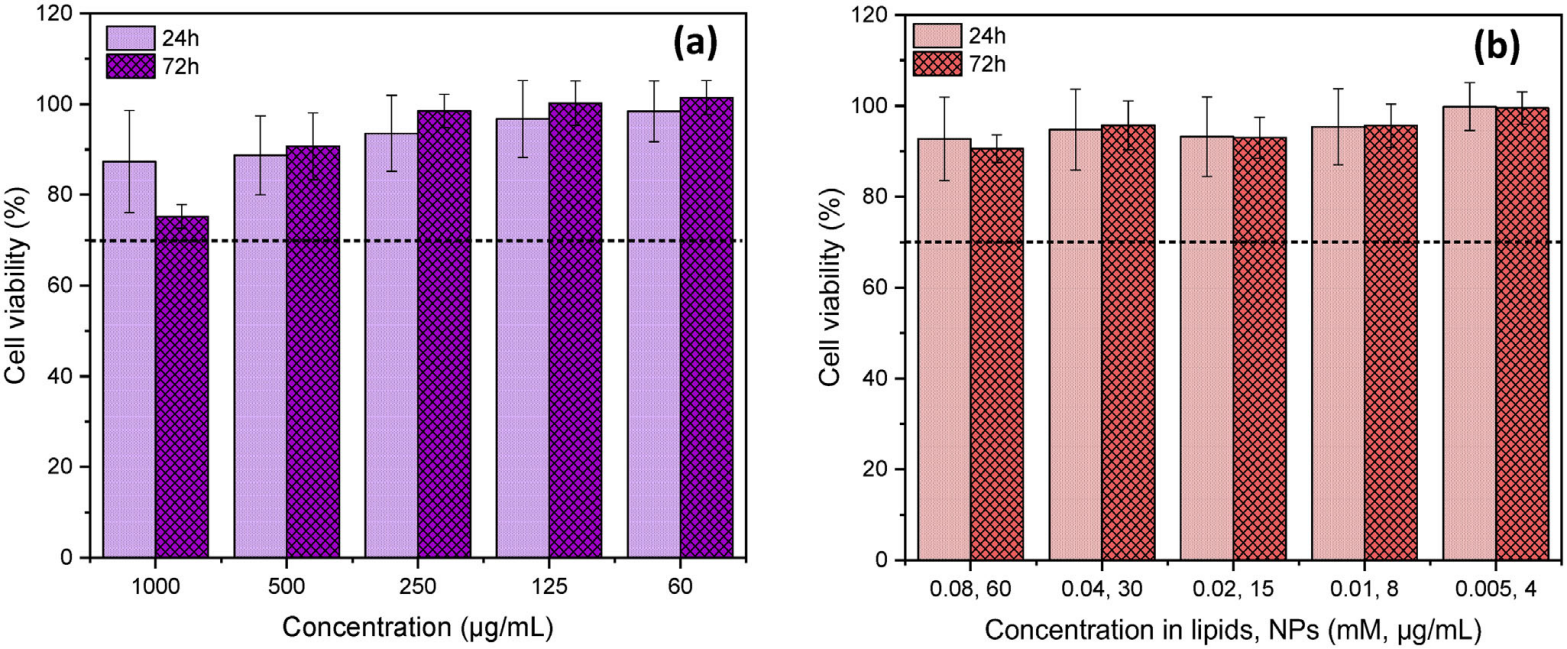
Canine fibroblasts viability evaluation after 24 and 72 h of incubation time at different concentrations against (a) Fe_3_O_4_:CA:CS NPs and (b) MLs. The black dashed line indicates the threshold for cell viability, according to the international standard procedure ISO 10993–5.

Results of cytotoxicity experiments are shown in Figure 9. As expected, the cytocompatibility of NPs was greatly enhanced by the CS coating (Figure 9a), especially at the highest concentrations (≥ 250 µg/mL) for which Fe_3_O_4_:CA NPs exhibited significant toxicity [53]. Free Fe_3_O_4_:CA:CS NPs were found non‐toxic even at a concentration of 1 mg/mL, which is largely above the ones commonly employed in clinical applications (∼50 µg/mL), such as MRI contrast agents [59, 60]. This was very interesting, as it showed that the addition of external Fe_3_O_4_:CA:CS NPs to enhance CF release from MLs was not detrimental to the biocompatibility. Also, liposomes were very well tolerated at concentrations up to 0.68 mm [53], and a similar biocompatibility was found for the MLs. While the MLs lipid membrane may protect cells from the relative toxicity of encapsulated Fe_3_O_4_:CA NPs, it has to be noted that it was not possible to increase the concentration of Fe_3_O_4_:CA NPs in MLs higher than 60 µg/mL (Figure 9b), a concentration for which they remained non‐toxic. This concentration limitation was due to losses during the purification process and sample dilutions (factor 10) in culture medium, necessary to perform the experiments. As a summary, the results presented here showed the biological relevance of the systems studied in this work, and allowed to envision future in vivo experiments.

## 4 Conclusion

We have successfully demonstrated the feasibility of a release of molecules from MLs, triggered by an external DC MF that can be applied in clinics within MRI devices. Fe_3_O_4_:CA NPs and Fe_3_O_4_:CA:CS NPs synthesized in this work were found to form necklace‐like nanoobjects and larger aggregated structures under a MF of *B*_0_ = 1.5 T, which corresponds to the most widely used MF strength in clinical MRI. Their structuration and its reversibility depended on several parameters, including NP type (*i.e*., presence or not of CS chains adsorbed in surface), *B*_0_ value, nature of the dispersion medium (water or carbonate buffer), and NP concentration. Fluorescence measurements were conducted to monitor CF release from liposomes and MLs suspended in carbonate buffer. Results evidenced a significant release from MLs loaded with 60 µg/mL of Fe_3_O_4_:CA NPs after a 25 min exposure to *B_0_* = 1.5 T. The addition of external Fe_3_O_4_:CA:CS NPs at a concentration of 1 mg/mL greatly enhanced the CF release, probably by strengthening the deformation/stress applied on the lipid membranes due to reciprocal interactions between encapsulated and external NPs. However, external NPs had no effect on empty liposomes. Finally, biological evaluation indicated that CS coating drastically improved the cytocompatibility of initial Fe_3_O_4_:CA NPs.

We believe that such results can have a major impact in the field of controlled drug delivery. Indeed, in contrast to AC MF‐driven release, the DC strategy provides a new route directly compatible with existing clinical MRI infrastructures and corresponding experimental protocols, thereby opening the way for drug release integrated within an MRI environment as well as for new theragnostic applications.

Future work will aim to define experimental parameters allowing to increase of the maximum amount of active substance that can be released, while enabling precise and tunable control over the delivered dose. These parameters include internal and external NP concentration, active substance content in MLs, use of other clinically relevant *B*_0_ strengths such as 0.5 T and 3 T, duration of *B*_0_ exposure, lipid composition, and temperature, among others. Finally, an in vivo study may be conducted to evaluate the biodistribution, stability, and capacity of these systems to deliver large quantities of active substance in real biological environments.

## Author Contributions

Jules Mistral contributed to investigation, conceptualization, methodology, and the writing of the original draft, as well as to writing, review, and editing. Nadège Milhau, Didier Pin, and Paula Nunes De Oliveira contributed to the investigation. Olivier Chapet contributed to funding acquisition. Anatoli Serghei and Guillaume Sudre contributed to supervision, with Guillaume Sudre also contributing to conceptualization, methodology, and the writing of the original draft. Catherine Ladavière and Laurent David contributed to funding acquisition, conceptualization, methodology, supervision, and the writing of the original draft, as well as to writing, review, and editing.

## Conflicts of Interest

There are no conflicts to declare. LD, CL, JM, GS, AS, OC have filed a patent entitled ″Suspensions de magnétoliposomes pour leur utilisation en tant que médicament sous champ magnétique permanent » submitted on 18, july 2025 n°FR2508278.

## Supporting information




**Supporting File 1**: smll72763‐sup‐0001‐SuppMat.docx.

**Supporting File 2**: smll72763‐sup‐0002‐Video.mp4.

**Supporting File 3**: smll72763‐sup‐0003‐Video.mp4.

## Data Availability

A CC‐BY public copyright license has been applied by the authors to the present document and will be applied to all subsequent versions up to the Author Accepted Manuscript arising from this submission, in accordance with the grant's open access conditions.
