# Supplementary material for: Clinically‐Relevant Static Magnetic Field Induces Release of Encapsulated Molecules from Magnetoliposomes
Source: Small. 2026 Mar 2;22(20):e11451. doi: 10.1002/smll.202511451 (PMC13054204; doi:10.1002/smll.202511451)
Supplement: Supplementary file 1 — Supporting File 1: smll72763‐sup‐0001‐SuppMat.docx. [file SMLL-22-e11451-s003.docx]

**Clinically-Relevant Static Magnetic Field Induces Release
of Encapsulated Molecules from Magnetoliposomes**

Jules Mistral,^a^ Nadège Milhau,^b^ Didier Pin,^b^ Olivier Chapet,^c^ Paula Nunes De Oliveira,^a^ Anatoli Serghei,^a^ Guillaume Sudre,^a^ Catherine Ladavière,^*a^ Laurent David,^*a^

^a^. Université Claude Bernard Lyon 1, INSA Lyon, Université Jean Monnet, CNRS UMR 5223, Ingénierie des Matériaux Polymères, F-69622 Cedex, Villeurbanne, France.

^b^. Veterinary School of Lyon (VetAgro Sup), UPSP 2016.A104 Interactions Cellules Environnement, F-69280 Cedex, Marcy l’Etoile, France.

^c^. Department of Radiation Oncology, CH Lyon-Sud, 165, Chemin du Grand Revoyet, F- 69310 Cedex, Pierre-Bénite, France.

* To whom correspondence should be addressed. [laurent.david@univ-lyon1.fr](mailto:laurent.david@univ-lyon1.fr) ; [catherine.ladaviere@univ-lyon1.fr](mailto:catherine.ladaviere@univ-lyon1.fr)

**Supporting information**

**SI1. TEM images and histograms of size distribution profile of Fe_3_O_4_:CA NPs.**

**SI2. Characteristics of various magnetic NP suspensions in aqueous medium.**

**SI3. Cryo-TEM images of MLs encapsulating Fe_3_O_4_:CA NPs, after purification by salt induced aggregation and magnetic chromatography.**

**SI4. WAXS characterization of the NP phase composition.**

**SI5. Experimental set-up for SAXS and WAXS measurements.**

**SI6. Stereo microscopy images of glass tubes before and after B_0_ exposure to magnetic field.**

**SI7. Images of glass tubes containing Fe_3_O_4_:CA NPs suspended in (a) water and (b) carbonate buffer (0.05 M, pH 9.2) at a NP concentration of 1 mg/mL, under B_0_ = 1.5 T.**

**S1. TEM images and histograms of size distribution profile of Fe_3_O_4_:CA NPs.**

The size profile was fitted with a log-normal law giving an average diameter d_TEM_ = 10.0 nm ± 2.2 nm.







**SI2. Characteristics of various magnetic NP suspensions in aqueous medium.**

| Sample | *D*_Z_ (nm) | PDI | ζ-potential (mV) |
| --- | --- | --- | --- |
| Fe_3_O_4_:CA | 65.8 ± 1.6 | 0.17 ± 0.01 | - 46.3 ± 14.3 |
| Fe_3_O_4_:CA:CS | 160.6 ± 2.6 | 0.14 ± 0.02 | + 38.1 ± 4.1 |

**SI3. Cryo-TEM images of MLs encapsulating Fe_3_O_4_:CA NPs, after purification by salt induced aggregation and magnetic chromatography.**

MLs are densely loaded with NPs, no external NPs outside MLs, as well as no empty liposomes can be observed, showing the efficiency of elaboration and purification processes.


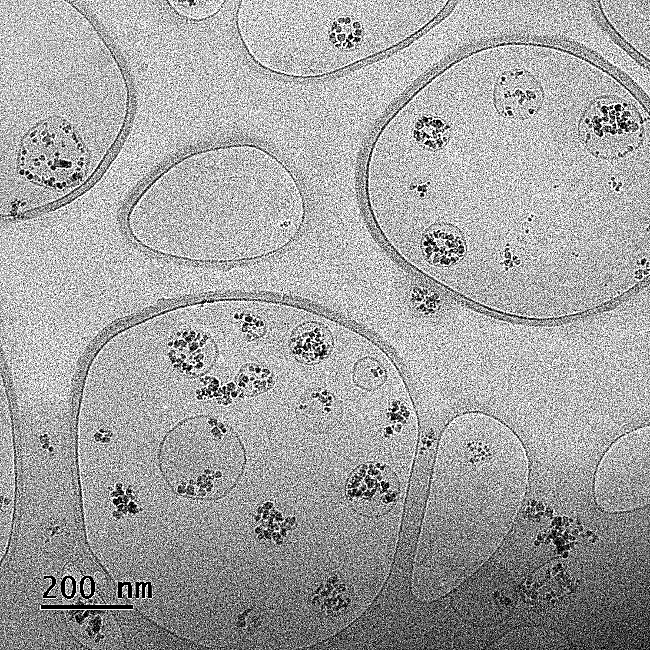

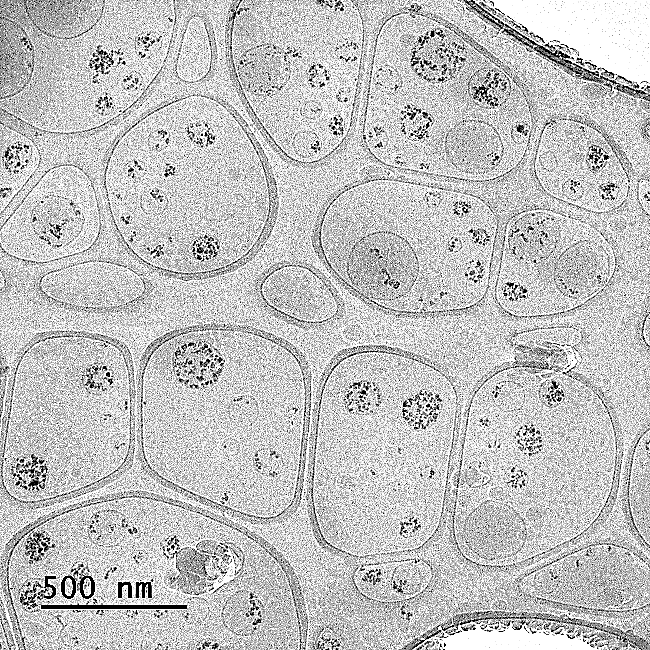


**SI4. WAXS characterization of the NP phase composition**

*The different images show WAXS diffraction diagrams (in transmission mode) obtained on NP powders for: (a) Fe_3_O_4_:CA NPs, (b) Fe_3_O_4_:CA:CS NPs.*

*On both graphs, the signal is largely dominated by the NP Fe_3_O_4_ cores. NP crystalline structure can clearly be identified with several peaks corresponding to (111), (220), (311), (222), (400), (422), (511), (440) family of planes of magnetite. No residual phases such as maghemite or hematite were detected.*

**(a)**

**(b)**

**SI5. Experimental set-up for SAXS and WAXS measurements.**

*The different images show: (a) home-made tube holder, to be inserted in the electromagnet device, (b) TE2M-50 electromagnet, with orange arrows indicating the zone of application of DC MF between the poles, (c-d) experimental set up on D2AM beamline in top-down views, with tube holder placed between the polar pieces of the electromagnet, allowing the glass tube to be in the DC MF zone, and submitted to the incident synchrotron beam.*


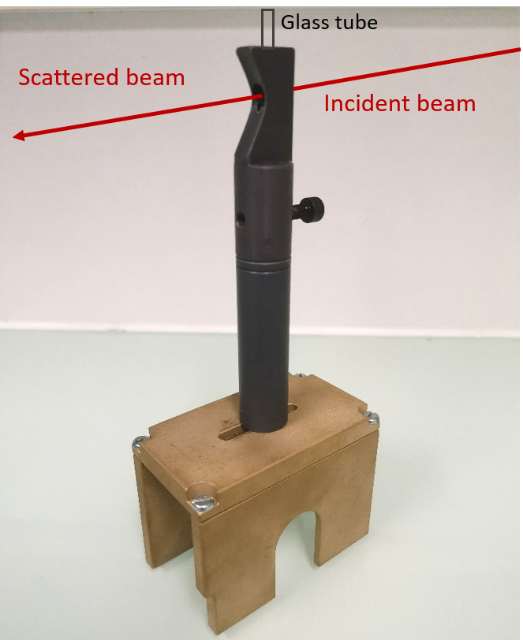


**(a)**


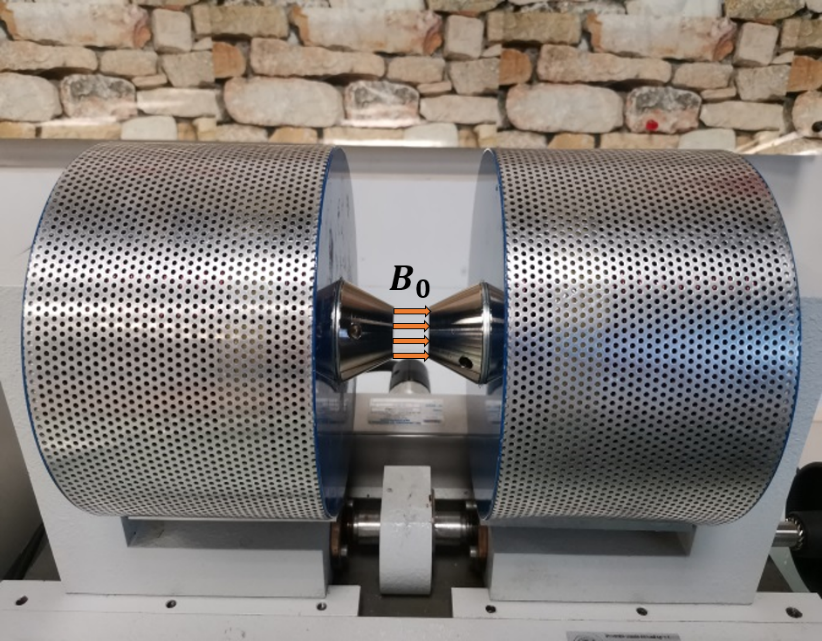


**(b)**


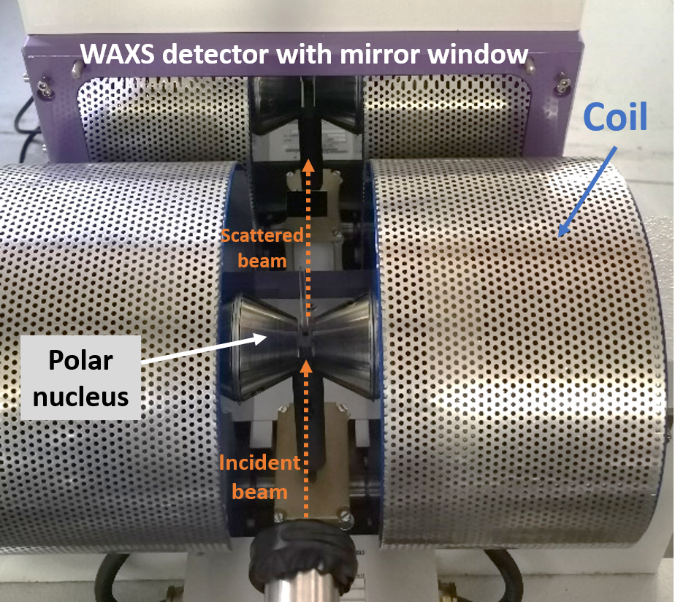


**(c)**


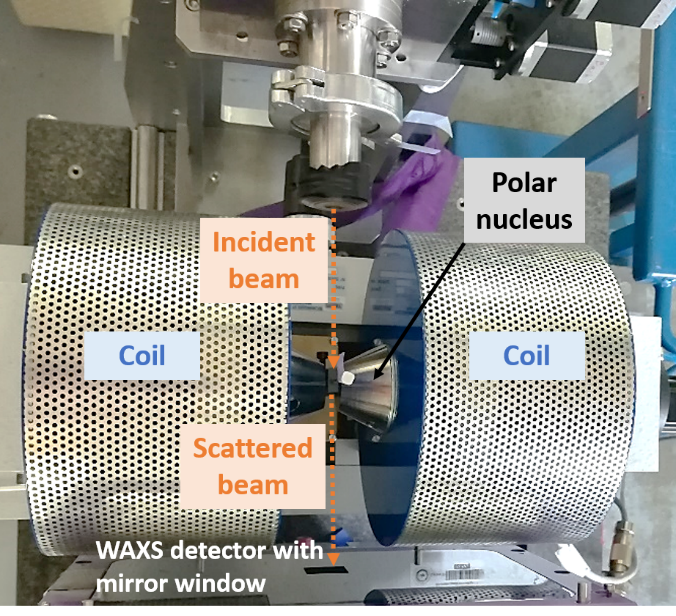


**(d)**

**SI6. Stereo microscopy images of glass tubes before and after B_0_ exposure to magnetic field.**

These tubes contain (a) Fe_3_O_4_:CA NPs before B_0_ exposure, (b) Fe_3_O_4_:CA NPs after B_0_ exposure to 1.5 T and sample removal from the magnetic field, (c) Fe_3_O_4_:CA:CS NPs before B_0_ exposure (d) Fe_3_O_4_:CA:CS NPs after B_0_ exposure to 1.5T and sample removal from the magnetic field. No residual structuration is observed for Fe_3_O_4_:CA NPs after B_0_ exposure, while a strong permanent structuration under the forms of fibrils can be seen for Fe_3_O_4_:CA:CS NPs.

***B*_0_**

***B*_0_**

***B*_0_**

***B*_0_**


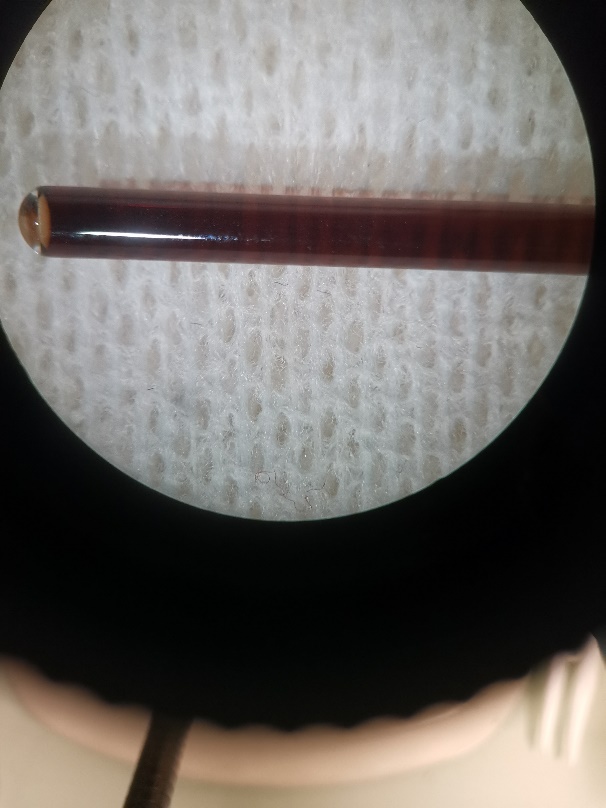


**(a)**


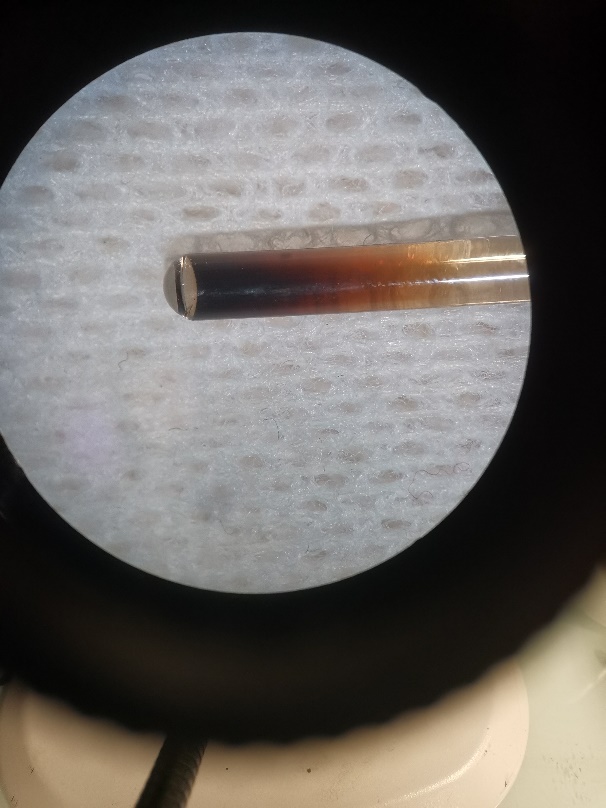


**(b)**


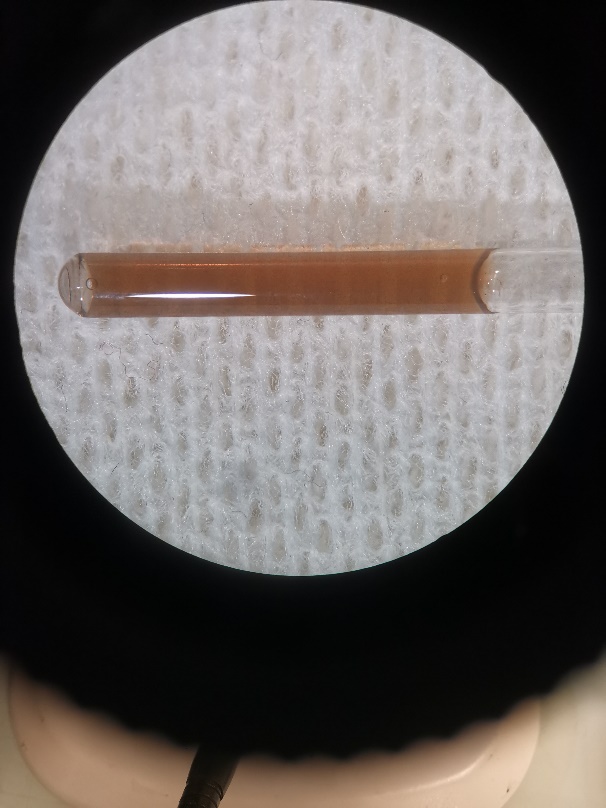


**(c)**


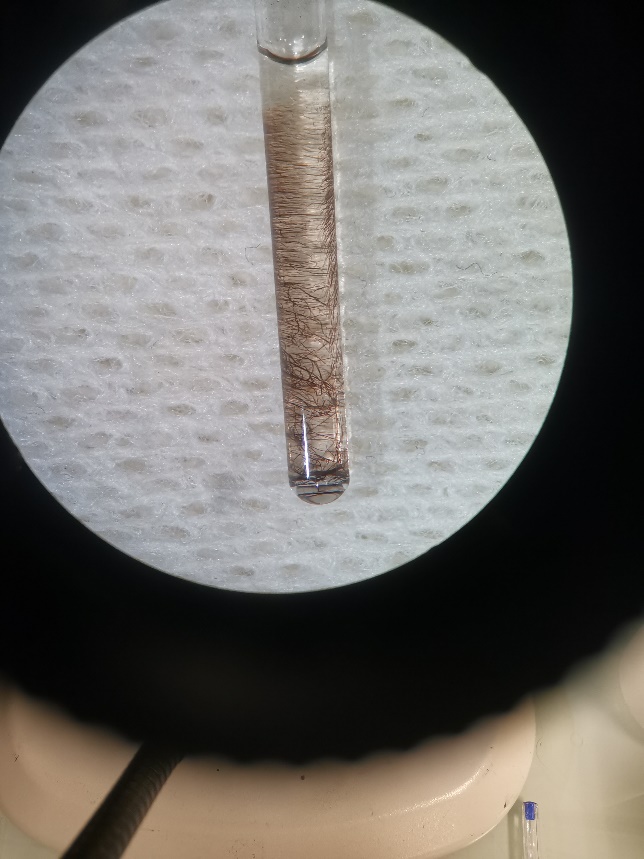


**(d)**

**SI7. Images of glass tubes containing Fe_3_O_4_:CA NPs suspended in (a) water and (b) carbonate buffer (0.05 M, pH 9.2) at a NP concentration of 1 mg/mL, under B_0_ = 1.5 T.**

Fe_3_O_4_:CA NPs show no visible structuration in water during B_0_ exposure. In carbonate buffer, a strong NP structuration under the form of fibrils aligned with B_0_ can be observed.


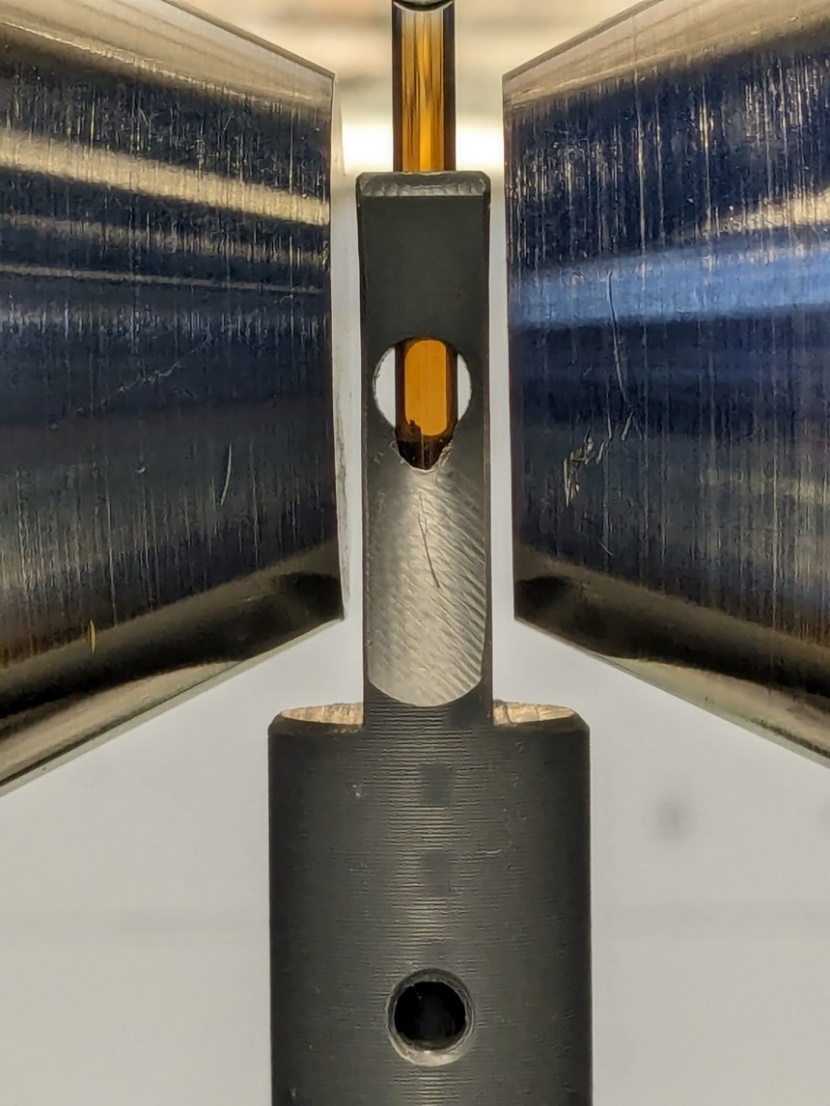


**(a)**


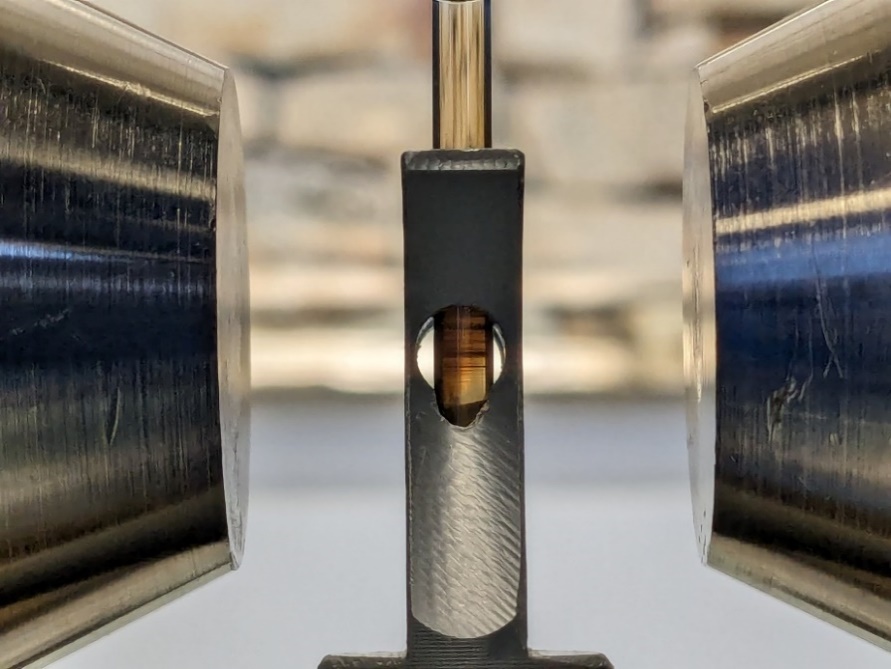


**(b)**
